# Right Femoral Fragility Fracture in an Adolescent with Vitamin D Deficiency from COVID-19 Pandemic-Related Confinement

**DOI:** 10.1155/2024/8354501

**Published:** 2024-03-11

**Authors:** Suhasheni Rajendran, Ze Chen Lee, Chu Ee Seow, Chong Hui Khaw

**Affiliations:** ^1^Department of Internal Medicine, Hospital Banting, Banting, Malaysia; ^2^Department of Internal Medicine, Hospital Kuala Lumpur, Kuala Lumpur, Malaysia; ^3^Department of Endocrinology, Hospital Pulau Pinang, George Town, Malaysia

## Abstract

**Background:**

The COVID-19 pandemic has caused major impacts in various aspects of our life. In Malaysia, a Movement Control Order was imposed in March 2020. For almost two years, school going children and adolescents were not able to attend school physically, and their physical activity was confined within their room or house on most days. *Case Description*. We describe a case of a 14-year-old boy who was previously active in sports and sustained a low trauma fracture at the right neck of the femur following a prolonged period of extreme sedentary life along with poor dietary intake during the COVID-19 pandemic period. He underwent open reduction and screw fixation for the right neck femur fracture. He was thin with a low BMI (15.62 kg/m^2^) and a significant loss of muscle bulk in all limbs. Laboratory tests showed vitamin D deficiency (15.3 nmol/L) and the dual energy X-ray absorptiometry (DXA) showed a low Z-score for the total spine (−2.2) and total hip (−3.9). He was treated with activated vitamin D and vitamin D3 replacement. Sports physician was involved for individualized postoperative rehabilitation. Successive clinic visits showed remarkable improvements in physical fitness, sports participation, and normalization of vitamin D levels.

**Conclusion:**

A high degree of suspicion is needed to rule out secondary causes in adolescents who present with unusual fragility fractures.

## 1. Introduction

Cutaneous synthesis from natural sun exposure is the primary source of vitamin D. Vitamin D deficiency reduces bone mass and increases the risk of osteoporotic fracture [1]. Movement Control Order (MCO) was commenced in Malaysia in March 2020 to combat the endemic spread of coronavirus disease 2019 (COVID-19). Lifestyle changes and dietary modifications during the COVID-19 lockdown were linked to overweight, nutritional deficiencies, reduced outdoor activity, and sun exposure.

## 2. Case Presentation

**Figure 1:**
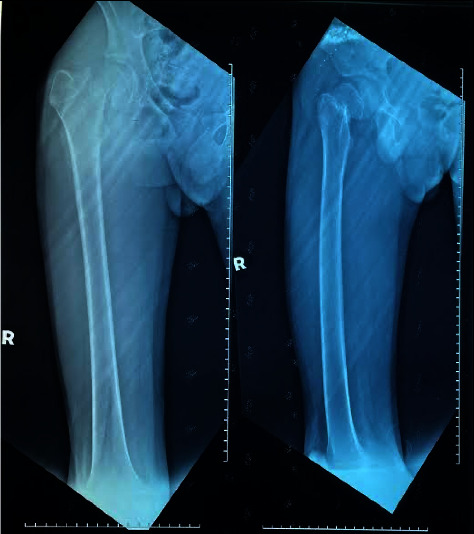
Right neck of femur fracture.

A 14-year-old boy with no prior medical history presented in October 2021 with a right femoral neck fracture (Figure 1) after slipping and falling from a standing position. He had no family history of bone or neuromuscular disease. He was active in outdoor cycling and soccer before the start of the Movement Control Order (MCO), but during the COVID-19 lockdown, he spent most of his time playing online games in a windowless room, limiting his sun exposure. His addiction to the mobile game led him to neglect his diet. Most of his meals during MCO comprised of fast food ordered via food delivery service, lacking of desirable nutrients including calcium and vitamin D. These lifestyle changes contributed to muscle loss and weight loss, without other constitutional or gastrointestinal symptoms.

He was assessed 6 weeks after the event at a visiting endocrinologist clinic. At the initial encounter, he was underweight, with a height of 158 cm, a weight of 38 kg, and a body mass index (BMI) of 15.62 kg/m^2^. His body weight was 42 kg with a BMI of 16.82 kg/m^2^ before MCO. He had marked muscle wasting, especially over his right thigh and both hands, but no obvious bony deformity over the spine or other long bones. He underwent an open reduction and internal fixation of his right femoral neck fracture. Investigations were performed to further evaluate the low-impact fracture (Table 1).

**Figure 2:**
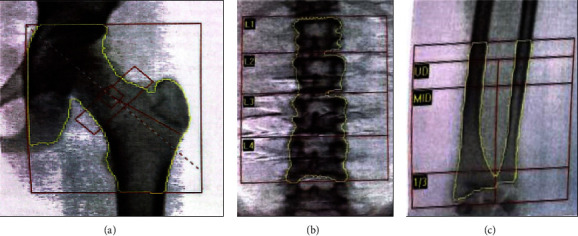
Dual energy X-ray images show low bone mineral density over (a) neck of femur, (b) lumber spine, and (c) distal radius.

A full blood picture showed normal erythrocyte morphology without evidence of blasts or abnormal lymphoid cell. Protein and red cells were absent in the urinary analysis. Antibodies for celiac disease were not sent, as he did not experience any gastrointestinal symptoms. He had severe vitamin D deficiency and significantly reduced bone mineral density (BMD) on dual-energy X-ray absorptiometry (DXA) (Figure 2), with low *Z*-scores at the lumbar spine (−2.2) and left hip (−3.9) as shown in Table 2. He was started on activated vitamin D alpha calcidiol 0.25 mcg and cholecalciferol 5000 IU daily.

After surgery, he was followed up in endocrine and sports medicine clinics. He underwent physiotherapy and was advised to increase his intake of dietary vitamin D and get adequate daily sun exposure. Four months later, his total vitamin D level had increased from 15.3 nmol/L to 62.15 nmol/L, and his DXA scan showed a marked improvement in *Z*-score (Table 2). Additionally, he showed a marked improvement in physical fitness, being able to jog and cycle during his recent endocrine clinic review, in contrast to a few months prior, when he had required crutches and a wheelchair for ambulation.

## 3. Discussion

Despite living in an equatorial climate with year-round sunshine, 30–50% of children and adolescents in Malaysia have vitamin D deficiency or insufficiency [2]. We report an unusual case of a low-energy right femoral fracture due to vitamin D deficiency in a 14-year-old boy with a sedentary lifestyle and reduced outdoor activity and sunlight exposure during the COVID-19 lockdown. Notably, lower vitamin D concentrations were also observed among children in China and Poland during the COVID-19 confinement period [3]. Other diseases associated with low bone mineral density were excluded through a thorough history and physical examination, as well as blood tests for neuromuscular disorders (e.g., Duchenne muscular dystrophy, myopathies), hematological diseases (e.g., leukemia, thalassemia), systemic autoimmune diseases (e.g., juvenile systemic lupus erythematosus, dermatomyositis, systemic sclerosis), gastrointestinal diseases (e.g., inflammatory bowel disease, celiac disease), renal disease (e.g., nephrotic syndrome, chronic kidney disease), and endocrine diseases (e.g., hypogonadism, hyperthyroidism, hyperparathyroidism, adrenal insufficiency, growth hormone deficiency). Muscle wasting noticed over hands and right thigh is most probably related to disuse atrophy, as physical examination did not detect other signs suggestive of neuromuscular disorders.

Unlike most adolescent fracture cases, which involve the humerus and distal forearm, our patient sustained a low-impact right femoral fracture after a fall from a standing position. This triggered the orthopedic team to refer him to rule out other possible causes of fragility fracture. His serum 25-hydroxyvitamin D level was critically low at 15.3 nmol/L (less than 30 nmol/L). His DXA scan showed a low *Z*-score at the lumbar spine and left hip. Ideally, bone mineral content should be measured over the lumbar spine and total body less head (TBLH) in adolescents to diagnose osteoporosis [4]. Clinical presentation in this case did not meet the criteria to define osteoporosis in adolescents, which require a BMD Z-score of ≤ −2.0 coupled with a clinically significant fracture history, including two or more long bone fractures by the age of 10 years, three or more long bone fractures at any age up to 19 years, or vertebral compression fractures [4]. Osteoporosis diagnosis criteria that require both a *Z*-score and a recurrent fracture may underdiagnose the condition, as patients may need to be monitored for a second or third fracture before diagnosis. Therefore, current recommendations advise considering clinical context in the diagnosis of osteoporosis, specifically the severity and prognosis of the underlying disease or treatment [5]. Lack of compensatory raise parathyroid hormone (PTH) level in this case is probable explained by hypomagnesemia and a decreased inhibitory threshold of PTH [6]. Alkaline phosphatase levels did not increase, as in most of the severe vitamin D deficiency cases. To date, studies on the relationships of alkaline phosphatase with serum vitamin D levels have shown variable results [6]. Hypophosphatasia carrier state could be an alternative explanation for his normal alkaline phosphatase, but urine phosphate levels and genetic testing were not performed [7].

The patient was started on activated vitamin D alpha calcidiol 0.25 mcg and cholecalciferol 5000 IU daily for 6 months duration, as per guidelines [6]. Multimodal management with the involvement of an orthopedic surgeon, endocrinologist, sports physician, and physiotherapist is crucial for subsequent management and rehabilitation. The patient showed marked gains in muscle bulks, improvements in physical fitness, serum vitamin D levels, and bone mineral density at succeeding follow-up sessions. The repeated calcium and phosphorus level subsequently were 2.42 mmol/L and 1.58 mmol/L, respectively. DXA will be repeated after one year and then every 1 to 2 years thereafter, depending on the patient's progression. The patient was also given dietary advice on a balanced diet that meets calcium and vitamin D requirements and was recommended to get adequate exposure to sunlight (at least six minutes per day) for optimal cutaneous synthesis of vitamin D [4, 8].

## 4. Conclusion

A high degree of suspicion is needed to rule out secondary causes in adolescents who present with unusual fragility fractures. Inadequate vitamin D bioavailability and physical activity reduce bone acquisition and peak bone mass, increasing the risk of low-impact fractures in teenagers. Early detection and intervention with sufficient calcium and vitamin D intake, adequate sunlight exposure, and maximized physical activity are paramount for maintaining bone health.

## Figures and Tables

**Table 1:** Lab investigations.

Lab	Result	Normal range
Calcium (corrected)	2.63 mmol/L	2.27–2.58 mmol/L
Phosphorus	1.67 mmol/L	0.78–1.65 mmol/L
Magnesium	0.48 mmol/L	0.65 to 1.05 mmol/L
25-hydroxyvitamin D	15.3 nmol/L	>30 nmol/L
iPTH	1.70 pmol/L	1.95–8.49 pmol/L
TSH	2.48 mU/L	
Free T4	21.3 pmol/L	
IGF-1	404.9 ng/ml	120–626 ng/ml
Testosterone	15.19 nmol/L	
FSH	3.9 IU/L	
LH	1.69 IU/L	
Random cortisol	115.8 nmol/L	
Total protein	74 g/L	
Albumin	47 g/L	
Globulin	27 g/L	
Alkaline phosphatase	194 U/L	115–471 U/L
Rheumatoid factor	Negative	
ANA	Equivocal	
C3	1.2 g/L	0.87–1.58 g/L
C4	0.22 g/L	0.14–0.36 g/L

iPTH intact parathyroid hormone, TSH thyroid-stimulating hormone, T4 thyroxine, IGF-1 insulin-like growth factor 1, LH luteinising hormone, FSH follicle stimulating hormone, ANA antinuclear Antibody.

**Table 2:** Dual energy X-ray absorptiometry.

Region	January 2022				December 2022		
	Area (cm^2^)	BMC (g)	BMD (g/cm^2^)	*Z*-score	Area (cm^2^)	BMC (g)	BMD (g/cm^2^)
Neck of femur	4.77	1.76	0.368	−4.4	5.12	2.70	0.527
Total hip	30.19	13.94	0.462	−3.9	34.68	20.73	0.598
Lumbar spine L1	9.14	4.92	0.538	−2.1	10.52	6.22	0.591
Lumbar spine L2	10.89	6.39	0.586	−2.1	11.91	8.12	0.681
Lumbar spine L3	12.54	7.30	0.582	−2.3	12.93	8.30	0.642
Lumbar spine L4	14.32	8.26	0.577	−2.2	15.18	9.86	0.650
Total L1–L4	46.90	26.86	0.573	−2.2	50.55	32.50	0.643
Distal 1/3 radius	1.01	0.22	0.217	NA	NA	NA	NA
